# Virtual screening of organic quinones as cathode materials for sodium-ion batteries†

**DOI:** 10.1039/d2ya00282e

**Published:** 2023-04-17

**Authors:** Xuan Zhou, René A. J. Janssen, Süleyman Er

**Affiliations:** a DIFFER – Dutch Institute for Fundamental Energy Research De Zaale 20 5612 AJ Eindhoven The Netherlands s.er@differ.nl; b Department of Applied Physics, Eindhoven University of Technology Eindhoven 5600 MB The Netherlands; c Molecular Materials and Nanosystems, Institute for Complex Molecular System, Eindhoven University of Technology Eindhoven 5600 MB The Netherlands

## Abstract

High-throughput virtual screening (HTVS) has been increasingly applied as an effective approach to find candidate materials for energy applications. We performed a HTVS study, which is powered by: (i) automated virtual screening library generation, (ii) automated search on a readily purchasable chemical space of quinone-based compounds, and (iii) computed physicochemical descriptors for the prediction of key battery-related features of compounds, including the reduction potential, gravimetric energy density, gravimetric charge capacity, and molecular stability. From the initial virtual library of approximately 450k molecules, a total of 326 compounds have been identified as commercially available. Among them, 289 of the molecules are predicted to be stable for the sodiation reactions that take place at the sodium-ion battery cathodes. To study the behaviour of molecules over time at room temperature, we performed molecular dynamics simulations on a group of sodiated product molecules, which was narrowed down to 21 quinones after scrutinizing the key battery performance indicators. As a result, 17 compounds are suggested for validation as candidate cathode materials in sodium-ion batteries.

## Abstract 2

The discovery of quinone-based compounds that are commercially accessible and can function as cathode materials in sodium ion batteries.

## Graphical abstract

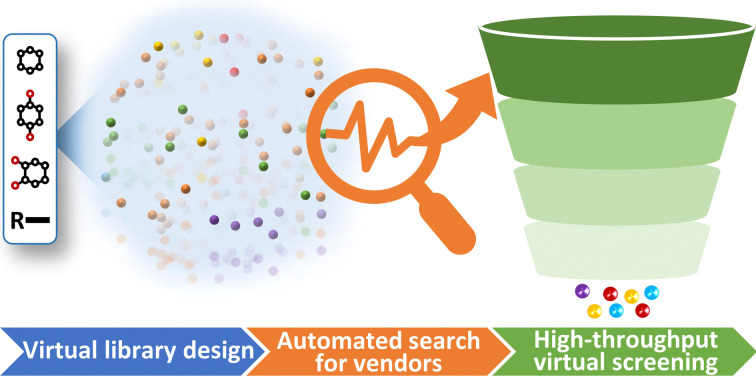

## 1 Introduction

Sodium-ion batteries (SIBs) attract increasing attention as promising alternatives to lithium-ion batteries (LIBs), because of the natural abundance, low cost, and high safety of sodium when compared to lithium as well as the similarities in the electrochemistry between SIBs and LIBs.^1–4^ An overwhelming majority of cathode materials in SIBs are inorganic compounds. They, however, suffer from the limitations of depletable raw material resources, large energy consumption during their synthesis, and structural deformations related to the embedding and stripping processes of the sodium ions.^5,6^ Driven by the demand for more sustainable and eco-friendly production and greater cost-effectiveness, organic electro-active compounds have been recommended as prime candidates of electro-active cathode materials in SIBs.^4^ Organic materials are renowned for their compositions based on earth-abundant elements, ease of processibility, and structural tailorability.^1,7^ Furthermore, the relatively flexible structural framework of organic materials induces less spatial hindrance during reversible insertion and extraction of sodium ions.^8^ Among all organic redox-active species, the family of quinones is one of the most widely researched electrode materials, as they show high energy capacity and fast redox-kinetics and yet can be profusely synthesized from biomass that will alleviate the environmental and economic costs of battery production.^9,10^

While both computational and experimental efforts have been increasingly devoted to the study of quinone-based cathode materials for SIBs,^10–14^ finding suitable electroactive compounds that are equipped with excellent electrochemical performance, which could eventually outcompete the conventional inorganic cathodes in some practical applications, is still a central topic of research. In the assessment of numerous organic-based electroactive candidates within a targeted chemical space, virtual screening is considered an effective strategy that saves time, resources, and costs, when contrasted with experimental trial-and-error approaches. Benefiting from the recent advances in computational techniques, workflows, and computing architectures, high-throughput virtual screening (HTVS) can speed up the identification of top-performing leads within a chemical search space. Recent HTVS efforts have demonstrated the effectiveness of this approach for the accelerated exploration of organic-based electroactive materials for batteries.^15–18^ Several important metrics that are related to the projected performance of the active materials can selectively and hierarchically be incorporated into the sifting stages of HTVS computational funnels.^17,19–21^ In this way, a workable number of candidate materials for the in-depth theoretical and experimental studies are down-selected from a vast screening space of chemical compounds in a notably reduced time window.

Deciding on a molecular search space serves as a starting point for the HTVS of quinones for SIBs. Instead of constructing a custom-made comprehensive virtual library on the fly, the effortless and immediate accessibility to existing quinones is of prime interest for HTVS, as then the candidate compounds are relatively free of the challenges in material synthesis and are readily accessible for rapid electrochemical testing under real lab conditions. The ZINC database is one of the largest open chemical databases with over 750 million purchasable molecules.^22^ Also important for the current work is that it provides stock information on the purchasable compounds that has been accumulated from various commercial vendors. Additionally, the database contains a large number of lightweight organic molecules that are likely redox-active, which are worthy of exploration. Here, our focus is on the computational chemical investigation of low molecular weight quinones with high charge capacity. In order to thoroughly cover the chemical space of existing quinones, we automatically searched the systematically created molecules in the ZINC database.

In the current study, a chemical library of organic quinones was created *in silico*, initially by combinatorial fusing of three different chemical building blocks and applying two design principles specifically for the number of carbonyl groups and ring structures and then by complete enumeration of the new derivatives of fused core skeletons with nine different R-groups. Meanwhile, for all the newly generated compounds, the commercial vendor information was automatically collected from the ZINC database. Thus, a total of 326 purchasable quinone molecules were identified and studied further through simulations. To predict the reduction potential of the candidate compounds for SIB cathodes, we developed a regression model based on the density functional theory (DFT)-calculated lowest unoccupied molecular orbital (LUMO) energies of the optimized pristine molecules and the experimentally measured reduction potential of quinone-based cathode materials in SIBs. Then, *via* HTVS involving DFT calculations, we optimized all 326 molecules in the gas phase and subsequently predicted their reduction potential, gravimetric charge capacity, and gravimetric energy density. In addition, we estimated the stability of the compounds using the calculated changes in molecular backbone atom positions, which are the consequence of sodiation redox reactions. By using the newly generated HTVS data on the battery performance-related descriptors, 21 quinone molecules were identified and their associated sodiated products were studied with molecular dynamics (MD) simulations. Consequently, 17 readily purchasable quinone compounds of diverse chemical motifs are recommended for further evaluation as potential cathode materials in SIBs.

## 2 Methods

### 2.1 Quantum chemistry calculations

All of the physics-based simulations were performed using the Schrödinger Materials Science Suite (SMSS).^23^ All the molecules were first optimized using the OPLS3e^24^ force field as implemented in the MacroModel^25^ package in order to find the lowest-energy 3D structure among the conformers of each molecule. Then, employing the Jaguar^26^ program, the B3LYP^27–29^ hybrid DFT functional together with the LACVP^++^**^30^ basis set with diffuse and polarization functions have been used for the further optimization of the molecular geometries and the calculation of properties. The convergence criteria for energy change and root mean squared (RMS) density matrix change were set at 5.0 × 10^−5^ and 5.0 × 10^−6^ hartree, respectively. A ‘medium’ grid density was used during structural optimizations, whereas a ‘fine’ grid density was applied during the consecutive single point energy calculations on the optimized geometries and the prediction of properties of the compounds. A similar calculation methodology and workflow have recently shown to be effective for predicting the reduction potentials of quinone-based cathode materials for LIBs.^31^

### 2.2 Molecular stability predictions

The (electro)chemical stability of cathode materials is an essential property that directly relates to the operational performance of SIBs. The stability can be quantified in experiments, for instance, by measuring the capacity retention after (dis)charge cycles. However, it is highly difficult to simulate the stability of organic-based cathodes, which is essentially due to lack of knowledge on the aggregated molecule structures and molecule crystals, as well as the (electro)chemical processes that may occur during (dis)charge cycles at electrode materials and interfaces. Therefore, frequently in HTVS, molecular-level calculations are resorted to in order to estimate the cycling stability of cathode materials. For this purpose, several descriptors, including the aromaticity of organic compounds,^32,33^ reorganization energy,^34^ and structural changes^19,34,35^ upon the reactions with lightweight alkali metal-ions have been considered as a proxy for the stability.

It has been previously reported that the changes in bond lengths of organic reactants over the (dis)charge processes are correlated to molecular stability, which eventually affects the kinetics of redox processes and rechargeability of electrodes.^34,36^ Therefore, organic compounds that undergo substantial structural changes upon their reaction with the metal-ions are not very likely to exhibit eminently reversible electrochemical behavior. In the current work, we compared the optimized structures of the reactant quinone molecules and their respective sodiated molecular products. By applying the LS-align algorithm,^37^ we calculated the root mean square deviation of carbon backbones (RMSD_C_) in atom coordinates between the optimized structures of pristine quinone molecules and their sodiated forms. In order to keep consistency in our comparisons throughout the diverse chemical space of the investigated quinone molecules, we only used the carbon atoms of the quinone backbones for the calculation of RMSD_C_, which are essentially by far the most prevalent chemical element, besides hydrogen, in the chemical composition of the quinone backbone structures.

### 2.3 Molecular dynamics simulations

MD simulations were carried out using the Desmond package,^38^ as implemented in SMSS. The OPLS all-atom^39,40^ force field was used for the MD simulations on product quinone molecules in a cubic box with an edge length of 50 Å. The system was modelled using the canonical ensemble (NVT),^41^ which had a temperature of 300 K and a pressure of 1 atm. To maintain the system's temperature, the Nose–Hoover chain thermostat^42^ method was used. The Coulombic interaction cut-off radius was set at 9 Å. The molecules were subjected to 100 ns of MD simulations each with a relaxation time of 1 ps. The behaviour of the molecules was analysed using the Simulation Interaction Diagram tool, with the MD simulation trajectories being recorded at 100 ps intervals.

To assess the stability of the molecules during the MD simulations, indicators such as the root mean square deviation of all atoms (RMSD_All_) and the root mean square fluctuation (RMSF) of heavy (*i.e.*, non-hydrogen) atoms in molecules were calculated and analysed. We used the RMSF data as a measure of structural flexibility of sodiated quinones, which is calculated using the below eqn (1):^43^1
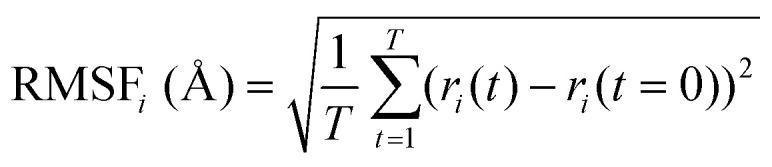
where *i* represents the index of the heavy atom in the compound, *T* is the total number of trajectory frames, and *r*_*i*_ (*t*) and *r*_*i*_ (*t* = 0) are the coordinates of atom *i* at time *t* and at the start of the MD simulations, respectively.

### 2.4 Calculation of charge capacity and energy density

The gravimetric charge capacity (*Q*), which describes the maximum number of charge carriers per unit mass of the cathode material, is expressed as:^13^2
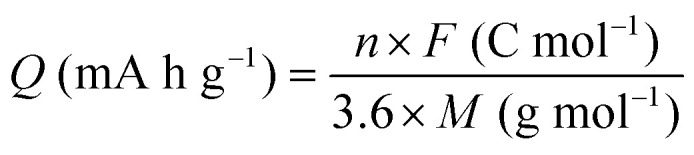
where *n* is the number of transferred electrons, *F* is the Faraday constant, and *M* is the molecular mass of the reactant quinone compound without the sodium atoms involved in redox reactions. Here, we assumed that all backbone carbonyl groups of the molecules can in principle be involved in the sodiation redox reactions. The molecular masses of reactant compounds are calculated using the simplified molecular input line entry system (SMILES) representations of molecules in the SMSS.

The gravimetric energy density (*W*), which describes the available energy per unit mass of the cathode material, is calculated as:^13^3
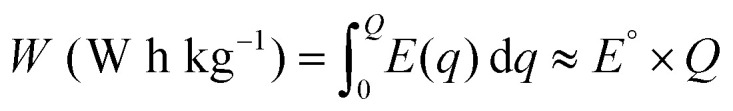
where *E*° represents the average reduction potential of the cathode material with respect to Na/Na^+^.

## 3 Results and discussion

### 3.1 Library generation and purchasable compound screening

**Fig. 1:**
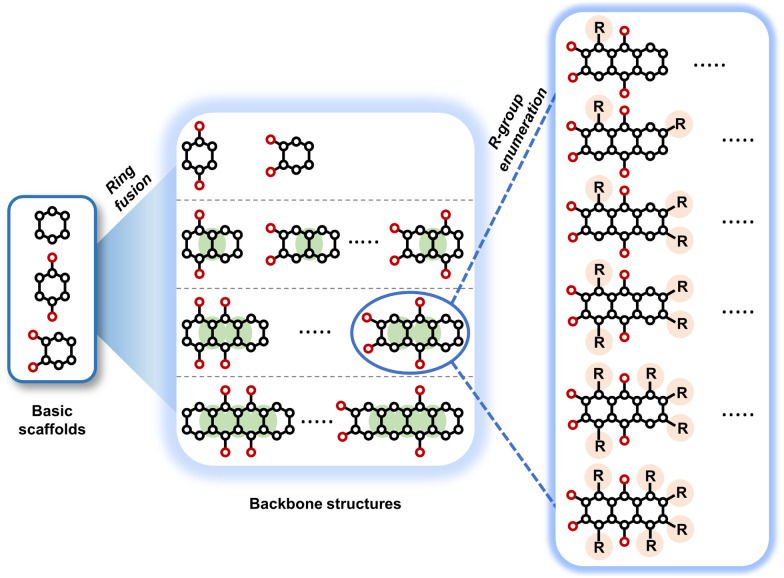
A schematic representation of the library generation process for the quinone-based compounds. The black and red spheres denote the carbon and oxygen atoms, respectively. The fused bonds over the ring fusion processes are shaded by green. The R-group substituted positions over the enumeration processes are shaded by orange.

To perform an exact structural search of purchasable compounds on the ZINC database, we first created a virtual library of small quinone-based compounds through a systematic approach as shown in Fig. 1. The backbone quinone structures have been generated by using three basic scaffolds, namely benzene, 1,4-benzoquinone, and 1,2-benzoquinone. The scaffolds have been fused together to populate the number of feasible backbone structures. Since we are interested in the quinone-based compounds here, a minimum of one 1,4-benzoquinone or 1,2-benzoquinone subunit was included for the fusion of ring structures. The intermolecular interactions, particularly due to the π–π stacking of molecules, increase as the size of aromatic compounds becomes larger.^44^ Thus, consequential computational deviations are expected when predicting the reduction potential of compounds as based on a single-molecule model. Therefore, to avoid large errors in the predicted reduction potentials of compounds when compared to the experimental measurements, we set limits for molecule size and number of redox-active sites on a molecule. Accordingly, we allowed a maximum of four rings and four carbonyl groups on the virtual molecules. As a result, a total of 170 unique quinone backbones were obtained with either of two or four carbonyl units, which is essentially due to the even number of carbonyls found in the 1,4-benzoquinone and 1,2-benzoquinone scaffolds. Next, to create R-group functionalized derivatives of the 170 backbone molecules, a systematic R-group enumeration routine has been applied. According to this, all the hydrogen atoms that are bonded to the molecular backbone carbon atoms have been consistently substituted with a particular R-group. A far-reaching effect in the electron-withdrawing and electron-donating ability of functional groups was intended for in order to induce local changes in the electron density on the redox-active carbonyl units of the quinone-based molecules.^45–49^ Thus, to cover a sizable chemical space when searching for redox active SIB cathode candidates, we considered nine different R-groups for the functionalization of all of the 170 backbone molecules. These R-groups were –CN, –CF_3_, –COOCH_3_, –F, –Cl, –Br, –CH_3_, –OCH_3_, and –NH_2_. The goal here is to optimize the performance of cathodes by modifying the quinone backbones with diverse R-groups, which alters the chemical composition, molecular structure, and electrochemical properties. This is achieved through a methodical approach of creating a virtual library of molecules with various functional groups, which increases both the chemical diversity and the size of the screening library. Moreover, the resulting molecular library serves as a point of reference when conducting precise structural searches on commercial vendor databases. Accordingly, a virtual screening library of 448 658 unique quinone-based chemically functionalized compounds has been created.

The commercial information of the newly created compounds has been tracked by using an in-house developed code, which performs an automated exact search of the molecular SMILES representations in the ZINC database and concurrently returns the recorded vendor information of the purchasable compounds from the database. After performing a search on the newly created virtual library of quinone-based compounds, we found a total of 326 molecules that were purchasable from at least one commercial vendor. A numerical summary of the virtual library creation process as well as the distribution of purchasable molecules with respect to the size of molecules is shown in Table 1.

**Table 1:** A summary of the systematically enumerated virtual chemical library of quinone-based small molecules and the commercially-available molecules that have been retrieved from the ZINC database. In addition to the total numbers of virtual and purchasable molecules in the library, the numbers of molecules as grouped per six-membered ring count within their backbone structures are also shown

Number of rings	Number of core structures	Maximum number of functionalized positions	Number of functionalized derivatives	Number of commercial molecules
1	2	4	137	78
2	9	6	1773	100
3	32	8	26 276	117
4	127	10	420 472	31
Total	170	—	448 658	326

### 3.2 Reduction potential predictions

**Fig. 2:**
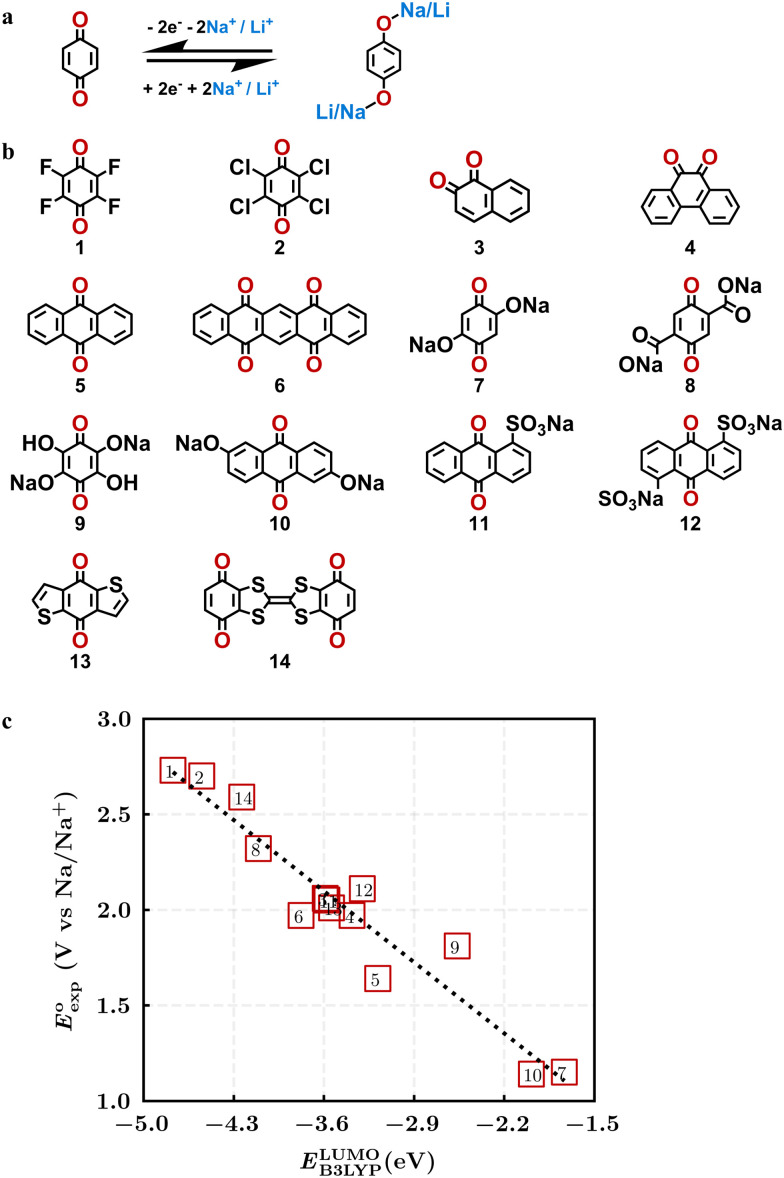
(a) A representation of the quinone reaction with metal ions at the cathodes of sodium- and lithium-ion batteries; (b) 2D structural representations of the fourteen experimentally studied quinones that have been used to develop an equation of calibration between the physicochemical descriptors and the reduction potentials; (c) observed performance of the DFT-optimized reactant molecule LUMO-energy as a descriptor for the prediction of measured reduction potentials in experiments.

Quinones show a similar reaction mechanism during both the lithiation and sodiation reactions.^50,51^ As shown in Fig. 2a, two metal-ion and electron pairs are involved for the reversible sodiation, or lithiation, of a quinone scaffold that contains two carbonyl units. It has been previously shown that the experimentally measured redox potentials of the LIB quinone cathode materials are most efficiently estimated by using the DFT-calculated gas phase LUMO energy of the reactant quinone molecules.^31^ Due to the analogy between lithiation and sodiation reactions of quinones, we also used here the LUMO energy of the pristine quinone molecules as a descriptor when predicting the redox potentials of SIB cathode materials.

In order to evaluate the performance of the LUMO-energy descriptor for the prediction of SIB cathode potentials, we collected experimentally measured redox data from the recent literature on quinone-based SIB cathode materials.^52–60^ The following criteria were applied to determine the inclusion of quinones in the calibration dataset:

1. Organic quinones should be the main component of the active materials in SIB cathodes.

2. When a quinone molecule has been studied in various research investigations, its experimental value is taken from a source that includes data on multiple quinones.

3. Quinones that have more than five rings are excluded. This is because as the number of aromatic rings increases, the π–π stacking interaction between the quinones increases at a fast rate,^44^ leading to greater inter-molecular interactions and higher inaccuracies in the gas phase approximation.

4. In order to maintain consistency with the digital molecular library design, each ring of quinones should contain at most a single pair of carbonyl groups, and the rings that possess carbonyl groups should be fused together.

Accordingly, we identified a total of 14 quinone-based molecules that are shown in Fig. 2b, which satisfy the above criteria and are included in the calibration dataset.

All of the selected quinones contain the redox-active substructures of either 1,4-benzoquinone or 1,2-benzoquinone. Moreover, they contain either one- or two-pairs of backbone carbonyl units. Furthermore, the number of backbone rings ranges from one to five. All of these features are of relevance to the structural diversity of the newly developed *in silico* screening library in the current study. As for the reference experimental reduction potential values, we used the average discharge voltage of the quinone cathodes, which had been obtained through the integration of charge–discharge curves. Accordingly, the experimental reduction potential of the quinones retained a voltage window of 1.59 V (Fig. 2c). Further information from experiments, including the employed organic electrolytes, average reduction potentials and measured discharge rates, are summarized in ESI,† Table S1.

The equation of a line of best fit, which correlates the experimentally measured reduction potential of the SIB quinone cathode materials to the DFT-calculated gas phase LUMO-energies of the quinone molecules, is given by:4
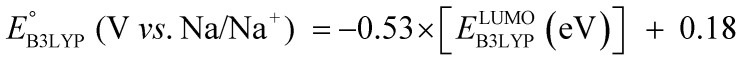
where 
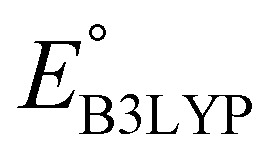
 is the predicted reduction potential at the B3LYP-level. The statistical measures, including the coefficient of determination (*R*^2^), root-mean-square error (RMSE), and normalized root-mean-square error (NRMSE), respectively, with the values of 0.92, 0.14 V, and 8.5%, indicate the fitness of the linear regression model. As a result, to predict the reduction potential of new quinone-based SIB cathode candidate materials, we employed the hybrid DFT-calculated gas-phase LUMO-energy of the optimized reactant molecules as shown in eqn (4).

### 3.3 HTVS of commercial quinones

In this section, we studied the library of 326 purchasable molecules in order to determine the most promising quinone-based molecules as SIB cathode materials. To prioritize the leading candidates, we first predicted the stability of the compounds by calculating the sodiation-induced structural changes of molecules in the gas-phase. In addition, we calculated the decisive battery-relevant metrics, including reduction potential, charge capacity, and energy density, all of which concern the practical use of the candidate cathode materials.

**Fig. 3:**
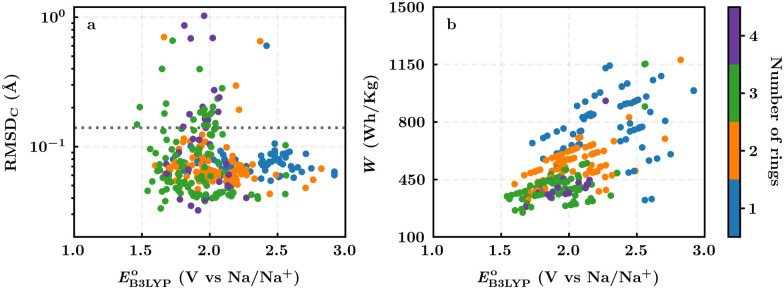
(a) A distribution of the calculated RMSD_C_ of the molecular structural changes upon sodiation reactions *versus* the predicted reduction potentials of the 326 commercially available quinones; (b) a distribution of the theoretical energy density *versus* the predicted reduction potential of the 289 quinones that are predicted to be stable during battery (dis)charge cycles. The different colors, as indicated by the color bar on the right, show the total number of six-membered rings that are present on the molecules.

For the complete set of 326 purchasable compounds, a distribution of the molecular geometry RMSD_C_ values over the sodiation process *versus* the DFT-predicted reduction potentials is shown in Fig. 3a. It is observed that the RMSD_C_ of molecular structures had no apparent correlation with the predicted reduction potential data of the molecules. Nevertheless, it is expected that a low RMSD_C_ would benefit rapid redox-kinetics and high cycling-stability for electroactive battery materials. Therefore, we applied a tolerance factor, *t*_b_, which is based on the change in bond lengths between the first-neighbor carbon atoms of the quinone backbones.^61^ According to this, we assumed that single (*d*_C–C_ ∼ 1.54 Å) and double (*d*_C=C_ ∼ 1.34 Å) bonds between two carbon atoms of the quinone backbones become aromatic at approximately 1.40 Å over sodiation reactions.^62^ Hence, any change in bond length that is greater than 0.14 Å indicates a weakening of the bonds beyond *t*_b_, which consequently would result in an increased probability of structural degradation over (dis)charge cycles. Accordingly, we applied *t*_b_ = 0.14 Å, which is indicated with a horizontal dotted line in Fig. 3a, for the calculated RMSD_C_ values between the DFT-optimized positions of carbon atoms in pristine and sodiated molecules. We assumed that molecules with small RMSD_C_ values would be stable over their redox reactions with sodium metal ions. Applying this criterion, 289 of the 326 purchasable quinones (*i.e.* 88% of the compounds) were found to have RMSD_C_ ≤ *t*_b_. A further analysis of the relationships between structural stability and cycling stability requires aggrandized volumes of experimental data on the aggregated structures of SIB cathode materials at their associated cycle numbers, which is currently lacking in the reported literature.

For the 289 quinones that are likely stable SIB cathode materials, a distribution of the theoretical gravimetric energy densities *versus* the predicted reduction potentials is shown in Fig. 3b. Both of the two electrochemical properties cover a wide range, which offers an abundant selection of candidates for the SIB cathodes and in relation to that expands the list of compliant SIB anode materials that could be employed at the counter electrode. As dictated by eqn (3) and shown in Fig. 3b, the quinones that feature very low reduction potentials are unlikely to embrace high energy densities, which once again stresses the importance of high reduction potentials as a must-have-property for the candidate cathode materials.

**Fig. 4:**
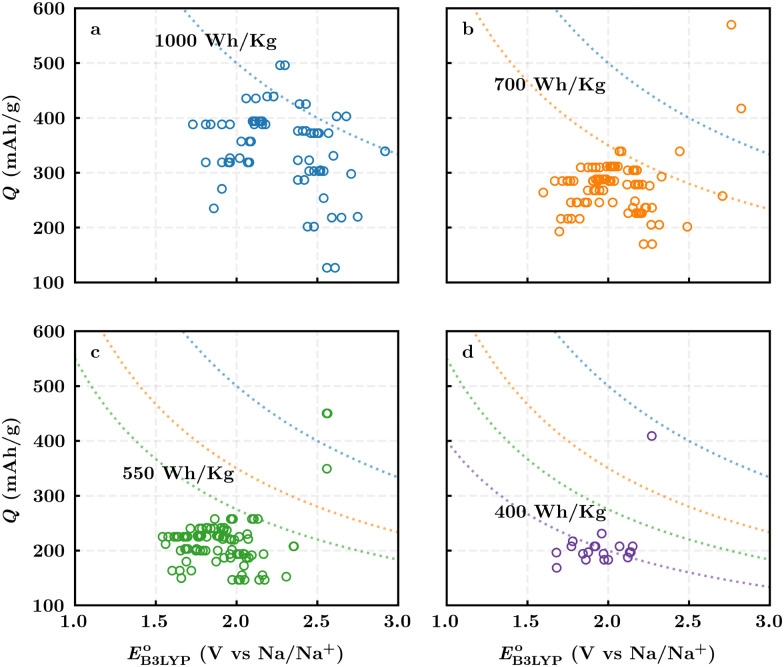
The distribution of the gravimetric charge capacity *versus* reduction potential for: (a) one-ring, (b) two-ring, (c) three-ring, and (d) four-ring compounds. Only the calculation data for 289 likely stable quinones, which have RMSD_C_ ≤ *t*_b_, are shown. The dotted blue, orange, green, and purple lines represent the cut-off energy density values for the group of molecules with increasing number of rings, respectively, from one to four.

Fig. 3b top-right includes the high-performance quinones as SIB cathode materials, which is heavily populated by relatively small (*e.g.*, one- or two-ring) compounds. However, low-molecular-weight quinones are prone to suffer from the undesirable dissolution in organic electrolytes, which for instance may be required for the preparation of cathode materials or the operation of SIB batteries. Therefore, when narrowing down the predicted list of top-performing compounds, it is also practically useful to consider the effect of molecular size, which may eventually relate to dissolution issues. For this reason, we grouped the candidates according to the total number of ring systems that are present in these molecules. Fig. 4 shows the distributions of gravimetric charge capacity *versus* reduction potential of quinones that have an increasing number of six-membered carbon rings in their structures. Thus, after grouping the molecules according to the number of rings that they have, there were 76 (26%) one-ring, 96 (33%) two-ring, 98 (34%) three-ring, and 19 (7%) four-ring molecules. In addition to the stability, reduction potential, gravimetric charge capacity and molecular size of compounds, the gravimetric energy density is an important property as discussed above. Therefore, we included in Fig. 4 some pragmatic guidelines, as shown with dotted lines, to illustrate the constant values for gravimetric energy density. As a result, we have identified a total of 21 quinones that possess favourable reduction potentials and energy density that meet the cut-off values for each group of molecules with various numbers of rings. The full list of these 21 molecules, including their basic structural representations and calculated electrochemical data, is shown in Table 2.

**Table 2:** Total number of rings, 2D structural representations, and the theoretical reduction potential 
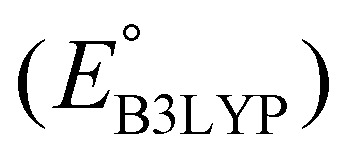
, gravimetric charge capacity (*Q*), gravimetric energy density (*W*), and InChIKey representation of the top 21 candidate quinones. In addition, average RMSD_All_ and RMSF_Max_ values of the associated sodiated products, as obtained by MD simulations, are shown

Ring count	2D structures	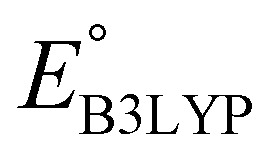 (V *vs*. Na/Na^+^)	*Q* (mA h g^−1^)	*W* (W h kg^−1^)	InChIKey	Average RMSD_All_ (Å)	RMSF_Max_ (Å)
1	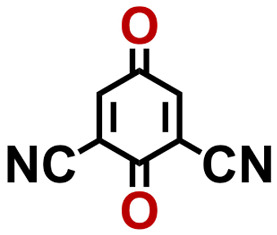	2.92	339	992	QNLVLMOZXQQZMB-UHFFFAOYSA-N	0.31	1.26
	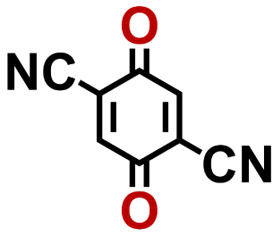	2.92	339	992	KIHAFRKCKZQZJX-UHFFFAOYSA-N	0.37	0.66
	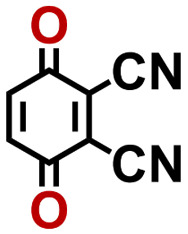	2.92	339	992	DNXUGBMARDFRGG-UHFFFAOYSA-N	0.46	0.86
	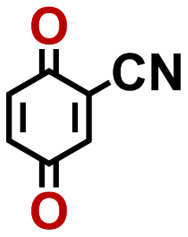	2.62	403	1054	RNKGDBXXIBUOTR-UHFFFAOYSA-N	0.33	0.81
	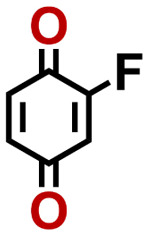	2.39	425	1015	XHKUTQNVGAHLPK-UHFFFAOYSA-N	0.30	0.58
	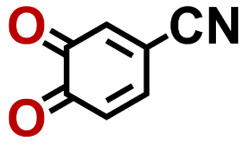	2.70	403	1079	YXBJVYOQFQQNAV-UHFFFAOYSA-N	0.67	0.21
	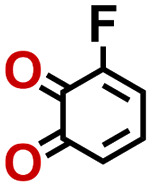	2.43	425	1034	UVVCKIWHWZKUKH-UHFFFAOYSA-N	1.48	0.16
	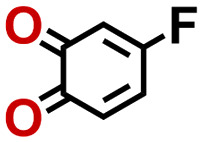	2.39	425	1016	KAAXOZWUPGCXMJ-UHFFFAOYSA-N	0.16	0.20
2	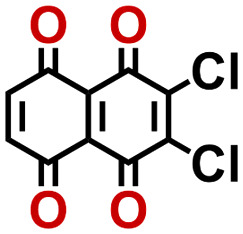	2.82	417	1178	JCGZOXQVXZJDSW-UHFFFAOYSA-N	0.32	0.64
	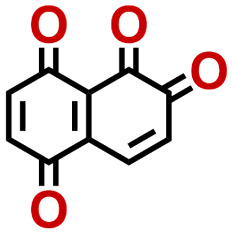	2.76	570	1574	YDRBIPYCZQHWPF-UHFFFAOYSA-N	0.54	1.37
	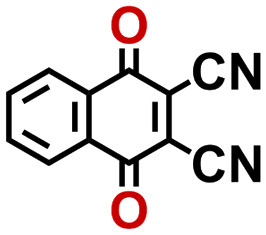	2.71	257	697	KIAJWKWOKTWTIZ-UHFFFAOYSA-N	0.44	0.95
	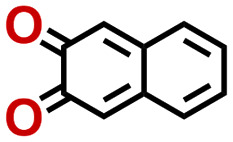	2.44	339	828	BBSQMTKECKKGCW-UHFFFAOYSA-N	0.16	0.23
3	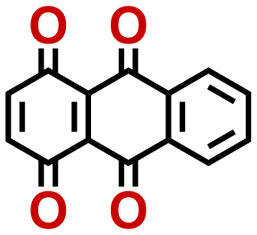	2.56	450	1154	KNCZUXHSQJQWIB-UHFFFAOYSA-N	0.31	0.71
	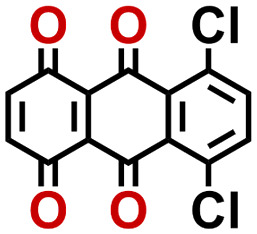	2.56	349	894	SCTWWYJJYPRROF-UHFFFAOYSA-N	0.32	0.87
	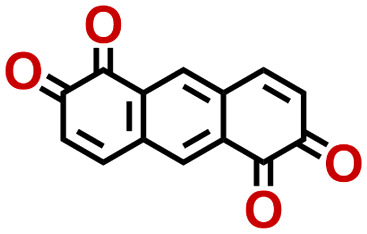	2.56	450	1151	CDZFYYHUDKHTEP-UHFFFAOYSA-N	0.26	0.61
4	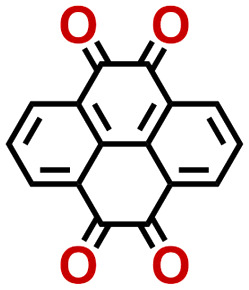	2.27	409	928	FQVOWPCGHLYSLB-UHFFFAOYSA-N	0.12	0.13
	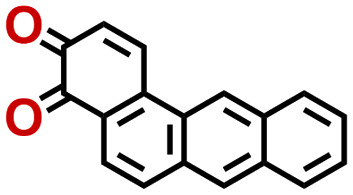	2.15	208	447	DABBKMJGXBSIAY-UHFFFAOYSA-N	0.17	0.31
	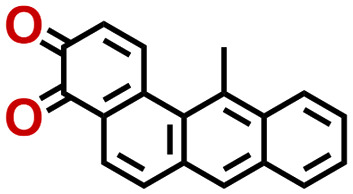	2.14	197	421	ZJJYEYXESLFXFZ-UHFFFAOYSA-N	0.32	0.80
	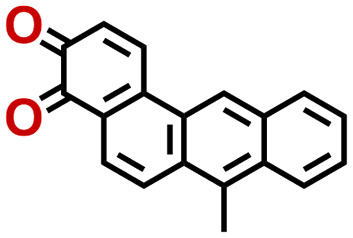	2.13	197	420	XUEZVDKAGOWVDJ-UHFFFAOYSA-N	0.19	0.32
	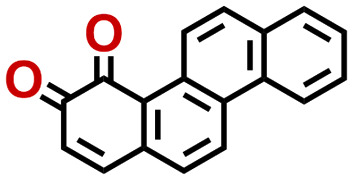	2.07	208	430	LLOUAGKAVSGKKD-UHFFFAOYSA-N	0.20	0.34
	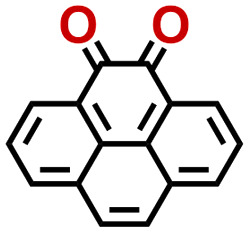	1.96	231	453	JKCATVQPENLMQJ-UHFFFAOYSA-N	0.88	0.22

To delve deeper into the stability of the 21 quinone molecules, MD simulations were carried out at 300 K and 1 atm pressure. For each molecule, the average value of RMSD_All_ and the highest value of RMSF (RMSF_Max_) are tabulated in Table 2, while the molecular trajectories and the atomic RMSF data are shown in Fig. S1 (ESI†). The molecular geometries and lengths of O–Na bonds both pre- and post-MD simulations are shown in Table S2 (ESI†). Notably, the seventh and twenty-first sodiated quinone molecules exhibit elevated average RMSD_All_ values (1.48 and 0.88 Å) despite having low RMSF_Max_ values (0.16 and 0.22 Å). This paradoxical pattern is a result of significant structural distortion in these two molecules, as evidenced by their perspective views at the end of MD calculations that are shown in Fig. S2 (ESI†). These findings suggest that the thermal stability of these two molecules is compromised following 100 ns of MD simulations. The 19 remaining sodiated quinone molecules exhibit relatively stable geometries, with average RMSD_All_ values below 0.67 Å. However, the flexibility of some of the O–Na bonds found in the first and tenth quinones in Table 2 is demonstrated by their high RMSF_Max_ values of 1.26 and 1.37 Å, respectively. Accordingly, a total of 17 sodiated molecules, which have both low average RMSD_All_ and RMSF_Max_ values that are within 1.00 Å, are found to be prime candidates for further validation studies.

The quinones show similar working principles in monovalent-ion batteries, in which their carbonyl groups interreact with the metal ions during the electrochemical (dis)charging reactions. Additionally, their crystal lattices have moderate softness, enabling compatibility with monovalent ions of various sizes. Consequently, the quinones identified as potential candidates for Na-ion batteries in this study also possess attractive qualities for use in other types of metal-ion batteries.

## 4 Conclusions

In the current study, we performed an automated exact structural search for a virtual molecular library of 448 658 quinone-based organic molecules in the chemical vendor databases. We found 326 electroactive molecules that were readily purchasable and practically testable in experiments with the aim of the discovery of new cathode materials for SIBs. To identify the most promising leads, we applied a DFT-empowered HTVS study that reckoned with the most essential battery-relevant properties of the compounds. A group of 21 quinones, which has not been previously tested as cathode materials in SIBs, were further subjected to MD simulations. Among them, 17 sodiated quinones exhibited low average RMSD values, making them ideal candidates for further validation studies.

## Author contributions

Conceptualization, S. E.; methodology, X. Z. and S. E.; modeling and validation, X. Z.; formal analysis and discussion, X. Z., R. A. J. J. and S. E.; writing – original draft preparation, X. Z.; writing – review and editing, X. Z., R. A. J. J. and S. E.; supervision, S. E.; project administration, S. E.; funding acquisition, X. Z. and S. E. All the authors have read and agreed to the published version of the manuscript.

## Conflicts of interest

There are no conflicts to declare.

## Supplementary Material

YA-002-D2YA00282E-s001
