# Supplementary material for: Virtual screening of organic quinones as cathode materials for sodium-ion batteries
Source: Energy Adv. 2023 Apr 17;2(6):820–8. doi: 10.1039/d2ya00282e (PMC10267898; doi:10.1039/d2ya00282e)
Supplement: YA-002-D2YA00282E-s001 [file YA-002-D2YA00282E-s001.pdf]

Electronic Supplementary Material (ESI) for Energy Advances.
This journal is © The Royal Society of Chemistry 2023

Supplementary Information

Virtual Screening of Organic Quinones as Cathode Materials for Sodium-Ion Batteries

Xuan Zhou,[a,b] René A.J. Janssen,[a,c] Süleyman Er, [a,*]

[a] *DIFFER – Dutch Institute for Fundamental Energy Research, De Zaale 20, Eindhoven, 5612 AJ, The Netherlands*

[b] *Department of Applied Physics, Eindhoven University of Technology, Eindhoven, 5600 MB, The Netherlands*

[c] *Molecular Materials and Nanosystems, Institute for Complex Molecular System, Eindhoven University of Technology, Eindhoven, 5600 MB, The Netherlands*

*Corresponding author

Email address: s.er@differ.nl (Süleyman Er,)

Table S1: A summary of key experimental data on molecules that have been used to develop an equation of calibration between the measured reduction potentials and the calculated LUMO energies.

**No.**	**2D structures**	**Organic electrolyte**	**Average reduction potential (V vs Na/Na^+)**	**Discharge condition**
1	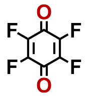	1 M $NaClO_4$/EC+PC+DMC (*v/v/v*=4.5:4.5:1)	2.73	10 mA/g
2	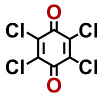	1 M $NaClO_4$/EC+PC+DMC (*v/v/v*=4.5:4.5:1)	2.70	10 mA/g
3	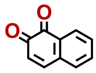	0.3 M NaTFSI/DME	2.06	34 mA/g
4	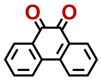	0.3 M NaTFSI/DME	1.96	26 mA/g
5	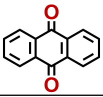	0.3 M NaTFSI/DME	1.64	26 mA/g
6	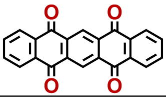	0.3 M NaTFSI/DME	1.97	20 mA/g
7	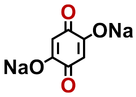	1 M $NaClO_4$/EC+DMC (*v/v*=1:1)	1.15	50 mA/g
8	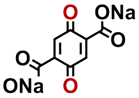	1 M $NaClO_4$/EC+DMC (*v/v*=1:1)	2.32	19 mA/g
9	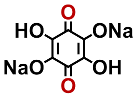	1 M $NaClO_4$/EC+DMC (*v/v*=1:1)	1.81	24.8 mA/g

Continued on next page

Table S1 – continued from previous page

No.	2D structures	Organic electrolyte	Average reduction potential (V vs Na/Na^{+})	Discharge condition
10	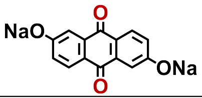	1 M $NaPF_6$/DME	1.14	50 mA/g
11	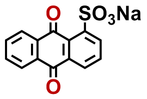	1 M $NaClO_4$/EC+DEC (*v/v*=1:1)	2.05	30 mA/g
12	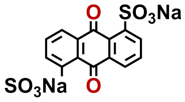	1 M $NaClO_4$/EC+DEC (*v/v*=1:1)	2.11	30 mA/g
13	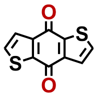	1 M $NaClO_4$/EC+DMC (*v/v*=1:1)	2.01	0.1 C
14	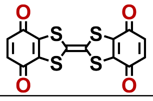	2.5 M NaTFSI/PC	2.59	40 mA/g

The abbreviations of organic solvents in the above table are denoted as: EC = ethylene carbonate, PC = propylene carbonate, DMC = dimethyl carbonate, DME= dimethyl ether, DEC = diethyl carbonate. The *v/v* indicates the volume fraction of organic solvents in a mixture.

Table S2: The initial and final geometries of the 21 sodiated product molecules and the bond distances between O and Na atoms ($d_{\text{O–Na}}$) as measured prior to and post MD simulations.

**Ring count**	**Initial geometries**	**Final geometries**	$\boldsymbol{d^{\text{Initial}}_{\text{O–Na}}}$ **(Å) (Top → Bottom)**	$\boldsymbol{d^{\text{Final}}_{\text{O–Na}}}$ **(Å) (Top → Bottom)**
1	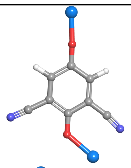	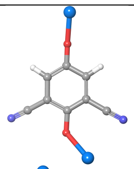	1.98<br>2.11	2.00<br>2.17
	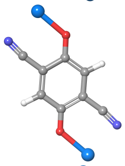	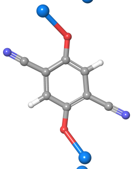	2.11<br>2.11	2.15<br>2.14
	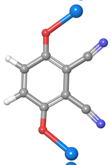	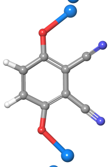	2.13<br>2.13	2.16<br>2.12
	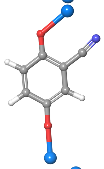	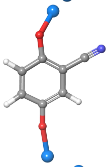	2.11<br>1.97	2.12<br>2.00
	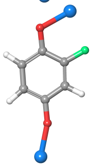	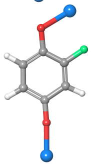	2.08<br>2.04	2.07<br>1.99
	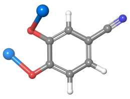	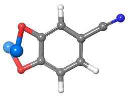	2.14<br>2.13	2.16<br>2.14
	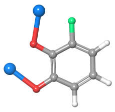	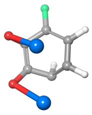	2.11<br>2.09	2.10<br>2.12
	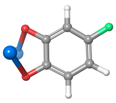	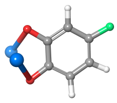	2.22<br>2.21	2.29<br>2.28

Continued on next page

Table S2 – continued from previous page

Ring count	Initial geometries	Final geometries	$d_{O–Na}^{Initial}$ ($\mathring{A}$) (Top → Bottom)	$d_{O–Na}^{Final}$ ($\mathring{A}$) (Top → Bottom)
2	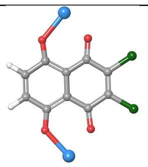	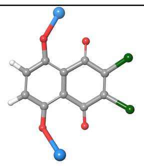	2.11<br>2.11	2.14<br>2.10
	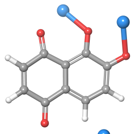	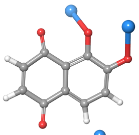	2.13<br>2.09	2.19<br>2.09
	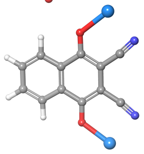	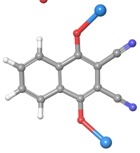	2.13<br>2.13	2.16<br>2.11
	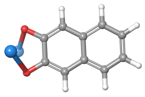	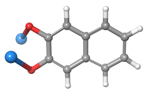	2.22<br>2.22	2.27<br>2.22
3	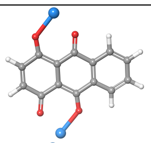	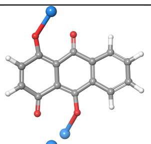	2.10<br>2.10	2.08<br>2.12
	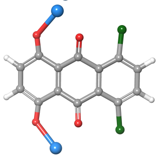	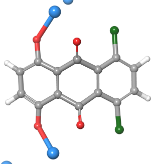	2.11<br>2.11	2.10<br>2.07
	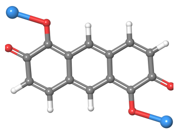	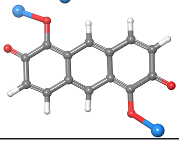	2.15<br>2.15	2.11<br>2.13

Continued on next page

Table S2 – continued from previous page

Ring count	Initial geometries	Final geometries	$d^{\mathrm{Initial}}_{\mathrm{O–Na}}$ (Å) (Top → Bottom)	$d^{\mathrm{Final}}_{\mathrm{O–Na}}$ (Å) (Top → Bottom)
4	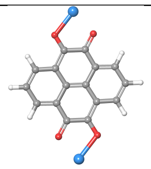	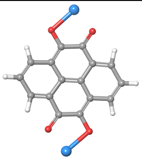	2.15<br>2.15	2.23<br>2.20
	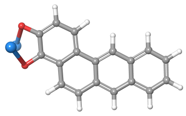	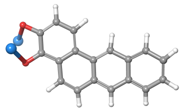	2.22<br>2.22	2.20<br>2.26
	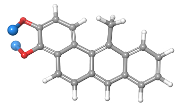	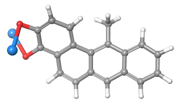	2.22<br>2.22	2.26<br>2.20
	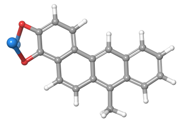	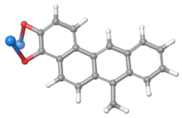	2.22<br>2.22	2.26<br>2.29
	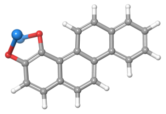	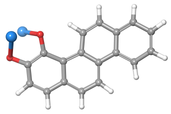	2.21<br>2.21	2.27<br>2.29
	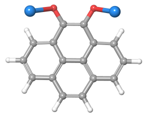	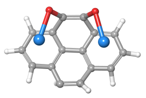	2.09<br>2.09	2.15<br>2.14

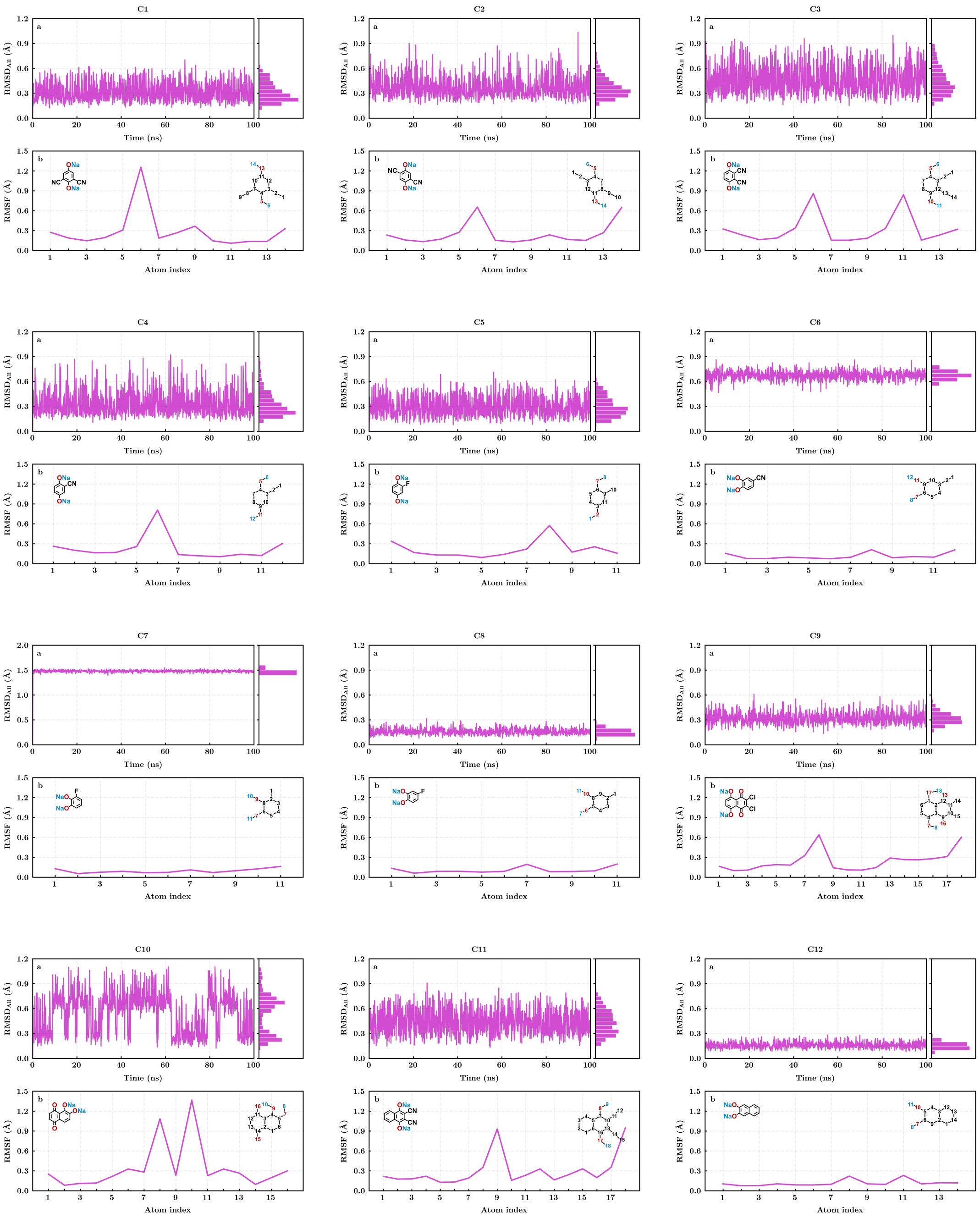
C1
C2
C3
C4
C5
C6
C7
C8
C9
C10
C11
C12
a
b
RMSD$_{All}$ (Å)
RMSF (Å)
Time (ns)
Atom index
ONa
NC
CN
NaO
F
Cl
O

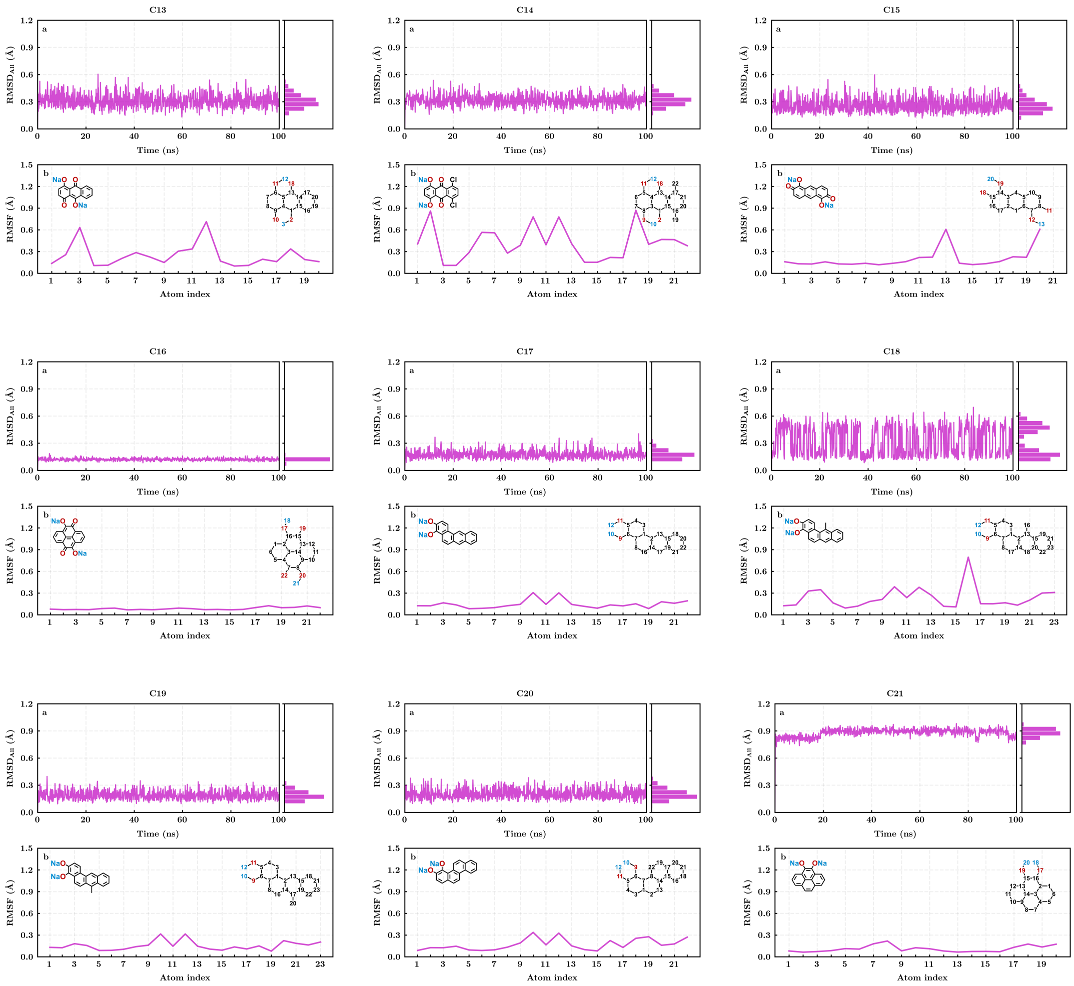

Figure S1: The MD simulation data of a total of 21 candidate (C) sodiated quinone molecules. For each molecule, (a) is the MD trajectory that shows the RMSD of all atoms in the sodiated product molecule, while the distribution of RMSD values is shown in the histogram on the right, and (b) is the root mean square fluctuation (RMSF) of the heavy atoms in the sodiated product molecule, where the top-left and top-right images represent the molecule's 2D structure and the atomic index of the heavy atoms, respectively. The C1-21 represent the 21 candidate molecules.

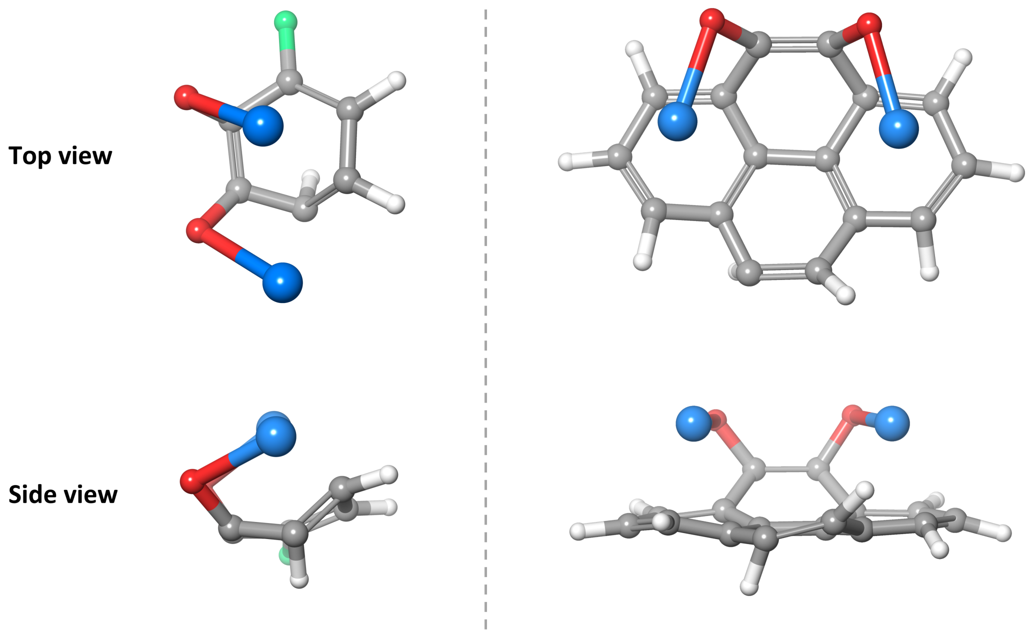

Figure S2: Top and side views of sodiated quinone geometries with high average $RMSD_{All}$ and low $RMSF_{Max}$ values.
